# Programmable directional color dynamics using plasmonics

**DOI:** 10.1038/s41378-023-00635-8

**Published:** 2024-02-01

**Authors:** Gyurin Kim, Doeun Kim, Soeun Ko, Jang-Hwan Han, Juhwan Kim, Joo Hwan Ko, Young Min Song, Hyeon-Ho Jeong

**Affiliations:** 1 School of Electrical Engineering and Computer Science, Gwangju Institute of Science and Technology, Gwangju, 61005 Republic of Korea; 2 Department of Semiconductor Engineering, Gwangju Institute of Science and Technology, Gwangju, 61005 Republic of Korea; 3 Artificial Intelligence (AI) Graduate School, Gwangju Institute of Science and Technology, Gwangju, 61005 Republic of Korea

**Keywords:** Nanophotonics and plasmonics, Nanophotonics and plasmonics, Nanoparticles

## Abstract

Adaptive multicolor filters have emerged as key components for ensuring color accuracy and resolution in outdoor visual devices. However, the current state of this technology is still in its infancy and largely reliant on liquid crystal devices that require high voltage and bulky structural designs. Here, we present a multicolor nanofilter consisting of multilayered ‘active’ plasmonic nanocomposites, wherein metallic nanoparticles are embedded within a conductive polymer nanofilm. These nanocomposites are fabricated with a total thickness below 100 nm using a ‘lithography-free’ method at the wafer level, and they inherently exhibit three prominent optical modes, accompanying scattering phenomena that produce distinct dichroic reflection and transmission colors. Here, a pivotal achievement is that all these colors are electrically manipulated with an applied external voltage of less than 1 V with 3.5 s of switching speed, encompassing the entire visible spectrum. Furthermore, this electrically programmable multicolor function enables the effective and dynamic modulation of the color temperature of white light across the warm-to-cool spectrum (3250 K–6250 K). This transformative capability is exceptionally valuable for enhancing the performance of outdoor optical devices that are independent of factors such as the sun’s elevation and prevailing weather conditions.

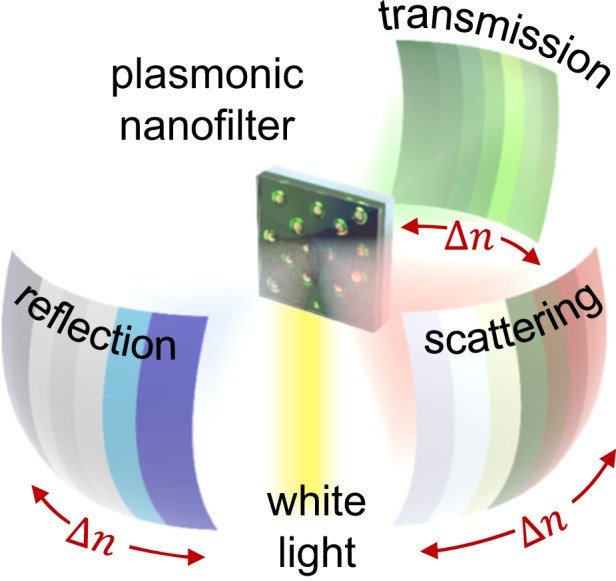

## Introduction

Human visual perception accounts for 85% of total information intake and plays a pivotal role in daily life^1^. This significance extends to imaging devices, which are essential for a wide spectrum of electronic applications ranging from personal devices such as mobile phones, tablets, and cameras to public systems such as closed-circuit television (CCTV) and self-driving vehicles. However, these imaging tools often have challenges with accurate object recognition in the face of fluctuating ambient light conditions, encompassing variations in brightness and color tone^2^. The realm of indoor lighting, for instance, spans a range of warm-to-cool color temperatures^3^, while outdoor sunlight’s spectral composition changes with daytime and nighttime shifts^4^, geographical location^5^, and prevailing weather conditions^6^.

To address these challenges, adaptive multicolor filters are emerging as indispensable tools, enabling tasks that demand real-time autocalibration of object colors under shifting light conditions^7,8^. Among the existing solutions, electrically tunable dichroic filters made of liquid crystals (LCs) have demonstrated color tunability across a wavelength range from 420 nm to 550 nm^9^. In these filters, the LC functions as a polarizer, selectively blocking specific wavelengths of light based on their alignment with an externally applied electric field^10^. However, these devices need high operational power (voltage > 6.5 V) to manipulate the microthick LCs (7 μm), thus causing a slow switching time exceeding 100 s and restricted color dynamics. For this reason, these LC-based systems have been improved with the aid of plasmonics, utilizing the strong light-matter interaction provided by subwavelength plasmonic nanostructures^11^. This approach has substantially reduced the thickness of devices to below 500 nm while achieving efficient full-color tuning with rapid switching times on the order of 1 s. Nevertheless, challenges persist, including the need for high power consumption to modulate the LCs (10 V) and the involvement of complex fabrication processes.

As an alternative, plasmonic metasurfaces integrated with conductive polymers have been demonstrated^12–15^. Conductive polymers envelop plasmonic resonators and manipulate colors by electrochemically altering their effective refractive indices^16^. For instance, the incorporation of a polyaniline shell around an array of silver nanocubes or gold nanorods with a thickness below 100 nm enables electrically tunable scattering with an input voltage of less than 1 V^17^. However, the sparse density of the plasmonic nanoparticles and their near-infrared resonances render them inadequate for multicolor filtering of visible light^18^. A recent advancement involves thin mirror-assisted plasmonic multilayers, where electrochromic nanoparticles reside on a thin mirror and cause the amplification of plasmonic scattering^19,20^. While this architecture generates electrically programmable scattering colors, providing both transmission and reflection color dynamics^21^, its color range remains limited due to the mirror, and the transmission color remains inadequate for comprehensive multicolor filtering.

Here, we present an electrically tunable dichroic multicolor nanofilter that only requires minimal electrical input (<1 V). This nanofilter device comprises metal-polymer nanocomposites featuring gold nanoparticles (Au NPs) embedded within a conductive polymer layer, specifically polyaniline (PANI), with a total thickness below 100 nm. This device utilizes the refractive index modulation of PANI under an applied potential, subsequently adjusting the resonance wavelength of the plasmonic Au NPs. This switching function leads to impressive color shifts of approximately 66 nm (green to red) in scattering, 42 nm (from blue to green) in reflection, and 89 nm (from blue to yellow) in transmission. This dynamic color range further facilitates the manipulation of the white light color temperature across the warm-to-cool spectrum (3250 K–6250 K) and has potential for tasks such as object color correction and recognition.

## Results

### Concept of electrically tunable dichroic multicolors using plasmonics

**Fig. 1:**
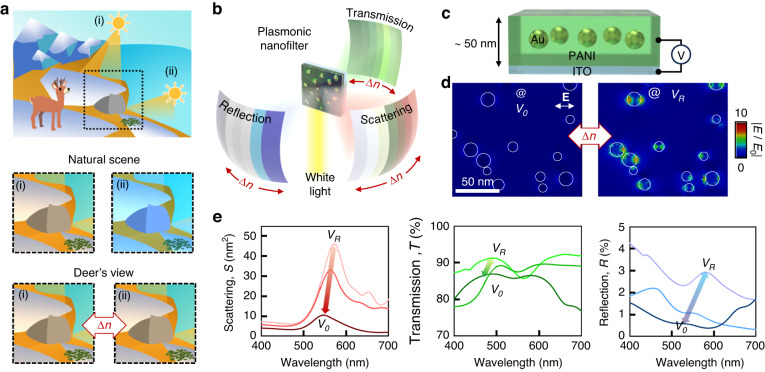
Programmable dichroic multicolors using plasmonics. **a** Concept of the reindeer eye’s adaptive coloration based on refractive index modulation. **b** Schematic of a programmable bidirectional color filter, which changes scattering, reflection, and transmission colors depending on the applied voltage. **c** Schematic of the plasmonic nanofilter and **d** associated optical near-field enhancements and **e** far-field scattering (left panel), transmission (middle panel), and reflection (right panel) when the applied voltage changes between ${V}_{R}$ (voltage for fully reduced PANI) and ${V}_{O}$ (voltage for fully oxidized PANI) from numerical simulations

Our inspiration arises from the adaptability of Arctic reindeer’s eyes to shift sunlight conditions (Fig. 1a)^22^. During the Arctic winter, the sun’s lower angle (−7.2°) nearly parallel to the surface results in a blue-biased sun irradiance spectrum due to specific light absorption properties of the ozone layer, termed the ‘Chappuis band’^23^. To counteract this phenomenon, the eye pressure of the Arctic reindeer adjusts through modification of the spacing between collagen fibril arrays in their eyes. This acts as a photonic crystal filter, modulating its effective refractive index $\varDelta n$ in response to pressure variations, and ultimately, the object colors become calibrated across diverse sunlight conditions. Here, we mimic this concept ($\varDelta n$ modulation) and create its plasmonic counterpart (Fig. 1b).

We apply the principle of localized surface plasmon resonance (LSPR), utilizing the scattering and absorption properties of subwavelength metal nanoparticles, generally with dimensions below 100 nm^24–28^. A key factor of this LSPR phenomenon is its susceptibility to color alteration in response to the refractive index of the nanoparticle’s surrounding medium $\varDelta n$^29^, reminiscent of the underlying modulation mechanism observed in the photonic structure of the deer eyes in Fig. 1a. While an active plasmonic color filter modulated by mechanical forces exists^30–32^, our focus is on refractive index modulation via an electrical approach, aligning with the requirements of commercial electrical devices. Among various responsive materials, including conductive polymers^33^ and phase change materials^34^, polyaniline (PANI) has emerged as a standout choice. Its appeal stems from significant refractive index tunability $\varDelta n$ ~ max. 0.6 within the visible range, facilitated by only a voltage input below 1 V^16^.

To apply this concept, we introduce plasmonic nanocomposites wherein gold nanoparticles (Au NPs) are randomly distributed within a PANI layer, resulting in an overall thickness of 55 nm (Fig. 1c). This design has a high density of monolayered Au NPs (effective mean diameter 11 nm) with a fill fraction of 25%, thereby promoting strong optical near-field enhancements (Fig. 1d) and far-field scattering signals (left panel of Fig. 1e). Importantly, our numerical simulation (Fig. S1) indicates an electrically switchable scattering color, transitioning from 551 nm at the oxidized PANI (${V}_{O}$) to 572 nm at the reduced PANI (${V}_{R}$). This alteration is attributed to the electrochemical redox reaction occurring within the PANI layer, which impacts the effective refractive index $\varDelta n$ surrounding the Au NPs. These dynamic scattering colors also affect the plasmonic extinction (sum of absorption and scattering), subsequently influencing the transmission and reflection colors along with their dynamics (middle and right panels of Fig. 1e). Under identical voltage inputs and consistent redox reactions, the transmission peak changes from 488 nm to 535 nm, while the reflection peak changes from 519 nm to 579 nm. We further validate via theoretical analysis that the PANI layer alone is not sufficient to induce scattering, yielding minimal modulation in reflection (from 400 nm to 452 nm) and transmission colors (from 479 nm to 512 nm) (Fig. S2A). Moreover, if the PANI layer partially covers the Au NPs, the color dynamics become less efficient (reflection from 399 nm to 456 nm and transmission from 504 nm to 541 nm, Fig. S2B). Therefore, the nanocomposite made of Au NPs embedded within the PANI layer facilitates bidirectional color dynamics, effectively spanning the wide visible range.

### Fabrication of the scalable plasmonic nanocomposites

**Fig. 2:**
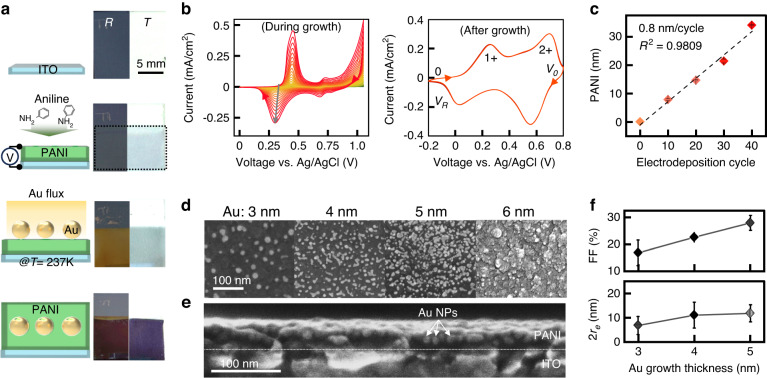
Fabrication of the plasmonic nanocomposites. **a** 3-step fabrication process, PANI electrodeposition on the ITO substrate, Au NP growth on top of the PANI layer, and PANI electrodeposition on Au NPs. **b** Cyclic voltammetry (CV) over 30 cycles of PANI electrodeposition (left panel) and corresponding CV (right panel) to confirm PANI’s redox reaction (0: fully reduced, ${V}_{R}$, 1 + : half oxidized, 2 + : fully oxidized, ${V}_{O}$). **c** Corresponding PANI layer thickness vs. electrodeposition cycle. **d** SEM images of the Au NPs on the PANI layer achieved from different growth thicknesses ranging from 3 nm to 6 nm with 1 nm intervals (top panel) and **e** cross-sectional images of a representative nanocomposite (bottom panel). **f** Fill fractions and sizes of the Au NPs *vs*. Au growth thickness

The fabrication process for this plasmonic nanofilter consists of a series of three sequential bottom-up growth steps, as shown in the left panels of Fig. 2a: (i) electrodeposition of PANI onto an indium tin oxide (ITO) substrate, (ii) physical growth of thin Au to yield Au NPs onto PANI, and (iii) subsequent electrodeposition of PANI to effectively encapsulate the Au NPs. This stepwise process not only produces the desired dichroic attributes at each stage, as evident in the right panels of Fig. 2a but also serves as the cornerstone for the subsequent color dynamics.

The sandwiched nanocomposite architecture produces dichroic colors through two pivotal engineering parameters. First, the thickness of the PANI layer on both the top and bottom facets of the Au NPs directly influences the tunability of the effective refractive index surrounding the Au NPs. Leveraging cyclic voltammetry for electrodeposition provides a convenient avenue for controlling this PANI thickness (Fig. 2b)^35^. Using voltage sweeps from negative to positive potentials enables the oxidation of anilines on the surface, followed by the incorporation of the free aniline monomers from the solution onto the surface, thereby forming an additional layer. Subsequent voltage reversal (from positive to negative) provides electrons to reduce all anilines, facilitating their polymerization (Fig. S3). This cyclic process results in a linear growth rate of the PANI layer at approximately 0.8 nm per cycle (Fig. 2c); this is estimated from their cyclic voltammetric curves (right panel of Fig. 2b) based on the charge and length of the aniline monomer (approximately 0.5 nm). To ensure uniform and stable PANI film deposition, the cycling number is limited to a maximum of 40 cycles, producing to an overall maximum thickness of 34 nm across the entire sample; this was determined by the stability and uniformity achieved using our in-house deposition setup.

The second significant engineering parameter is the geometry (i.e., size and fill fraction) of the Au NPs, which fundamentally determines the peak wavelength of the LSPR^36–38^. To create Au NPs on the PANI layer, we adopted a physical vacuum growth method combined with substrate cooling. Cooling the substrate at 237 K serves a dual purpose: it prevents the PANI layer from thermal damage caused by Au adatoms upon landing and concurrently suppresses the diffusion of Au adatoms, promoting the uniform growth of Au NPs^39^. We systematically grow the Au films in increments of 1 nm, spanning from 3 nm to 6 nm, closely monitored through a quartz crystal microbalance (QCM) system (Fig. 2d). Morphologically, the Au NPs show two primary configurations: particle-like formations, encompassing monomers and multimers, and a continuous film-like morphology. The prevalence of particle-like morphology is particular for thicknesses ranging from 3 to 5 nm, displaying an incremental increase in the fill fraction from 17% to 28% while maintaining a saturation of particle size ca. 11 nm with a maximum ±8 nm size variation (Figs. 2f and S4). In contrast, the film-like morphology predominates when the Au growth thickness exceeds 6 nm, which is undesirable for LSPR applications. As a result, we constrain the maximum growth thickness to 5 nm to preserve the intended characteristics of the LSPR phenomenon. Notably, unlike nanoparticle coating methods from colloidal solution^40^, this direct physical growth method provides significant flexibility in the choice of nanoparticle materials and enables systematic control over their structural morphology, including size and number density. This method has potential for the exploration of a wide range of plasmonic materials, including but not limited to Ag^41^, Cu^42^, Al^38^, and their alloys^43,44^, thereby extending its applicability to various wavelength regimes.

### Electrically tunable dichroic multicolors from the plasmonic nanocomposites

**Fig. 3:**
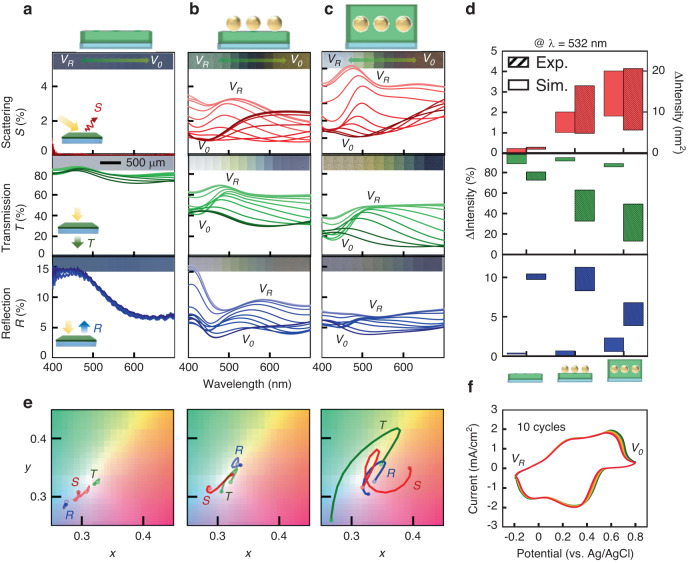
Electrically tunable dichroic multicolors. Experimentally measured color dynamics of *S*, scattering (top), *T*, transmission (middle), and *R*, reflection (bottom) of **a** as-grown 21 nm PANI film, **b** Au NPs with a size of 11 nm on the PANI film, and **c** 8 nm PANI covered on Au NPs when the voltage is applied from −0.2 V (${V}_{R}$) to 0.8 V (${V}_{O}$). Each inset shows the corresponding optical images. **d** Intensity changes under the green light (532 nm) of each mode for each fabricated structure and **e** associated color gamut plots (CIE 1931 chromaticity). **f** Cyclic voltammetry (CV) with over 10 cycles of the device while measuring the optical properties

To comprehensively measure and analyze the dichroic properties of the plasmonic nanocomposites, we integrate the sample within a compact electrochemical cell. This configuration enables simultaneous real-time measurements of optical spectra and images while applying dynamic potential through a potentiostat (Fig. S5). This approach enables the collection of the scattering, transmission, and reflection spectra, along with their associated optical images taken during electrical tuning (Fig. 3 and Video S1). Notably, the PANI layer itself lacks scattering capabilities, as evidenced by the absence of scattering signals from an approximately 21 nm thick PANI film (top panel of Fig. 3a). Consequently, only marginal adjustments are observed in transmission (4% change) and reflection (1% change), yielding trivial color dynamics (transmission range of 459–477 nm and reflection range of 451–456 nm), middle and bottom panels of Fig. 3a. These alterations are attributed to the change in the effective refractive index of the PANI layer.

However, when Au NPs with a size of 11 nm with a 25% fill fraction are introduced on top of this PANI layer, plasmonic scattering signals emerge, resulting in dynamic color tuning spanning from 456 nm to 517 nm, accompanied by a 2.4% intensity change (top panel of Fig. 3b). This, in turn, leads to expanded multicolor tunability in both transmission and reflection modes (middle and bottom panels of Fig. 3b). The transmission color shifts from 436 nm to 501 nm, spanning colors from white to blue, and the reflection color transitions from 526 nm to 589 nm, showing colors from yellow to green. The complete encapsulation of Au NPs within the PANI layer facilitates efficient modulation of the LSPR signals, consequently yielding scattering spectra with peak wavelengths spanning 426 nm to 493 nm, accompanied by 4% intensity tuning at resonance (top panel of Fig. 3c). This enhanced scattering tunability further extends the color tuning in the transmission wavelengths from 432 nm to 521 nm, along with 37% optical contrast at resonance, and in the reflection wavelengths from 450 nm to 492 nm with 3.2% optical contrast (Fig. 3c, d). Importantly, the colors depicted by both spectra and images closely align in the CIE 1931 color space, effectively demonstrating the pronounced expansion of the color dynamics when plasmonic Au NPs are fully integrated and encapsulated within the PANI layer (Fig. 3e). We also confirm that since the Au NPs are randomly distributed with various sizes, their associated color dynamics are rather insensitive to the growth thickness of the Au film; thus, highly reproducible color dynamics can be achieved (Fig. S6). As a result, the singular plasmonic nanocomposite with mode-switching capabilities shows a full-color transition from yellow to blue in transmission mode, white to red in scattering mode, and green to blue in reflection mode based on the stable electrochemical reaction of the PANI layer (Fig. 3f).

**Fig. 4:**
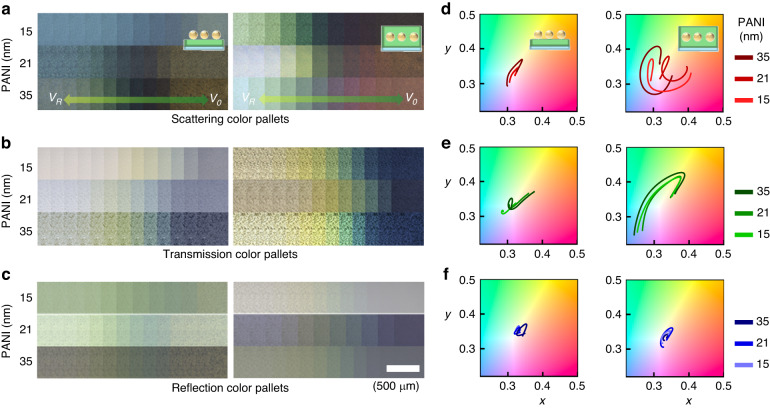
Programmable multicolor modulations with PANI thickness engineering. **a** Scattering, **b** transmission, and **c** reflection images of the plasmonic nanocomposites with various PANI conditions. Associated **d** scattering, **e** transmission, and **f** reflection CIE plots

Furthermore, we engineer the color dynamics of our plasmonic nanocomposites by manipulating the geometry of the PANI layer (Figs. 4, S7 and Video S2). Our approach involves two intuitive strategies, controlling the coverage of the PANI layer around the Au NPs (as observed in Fig. 3) and adjusting its thickness. Here, we strategically vary both parameters. The lower PANI layer has thickness values of 15 nm, 21 nm, and 35 nm, while the upper layer is either absent (left panels of Fig. 4a–c) or present with a thickness of ca. 8 nm (right panels of Fig. 4a–c). These diverse configurations produce electrically tunable colorful dynamics, perceptible in scattering (Fig. 4a), transmission (Fig. 4b), and reflection modes (Fig. 4c). Across all thickness variations of the PANI layer, the plasmonic composites involving an upper PANI layer consistently show notably wider color modulations across all optical modes while visualizing different color dynamic ranges compared to their counterparts lacking this supplementary layer (Fig. 4d–f). This divergence is attributed to the influence of the upper PANI layer on the modulation of the effective refractive index $\varDelta n$ surrounding the Au NPs, thereby intricately adjusting the corresponding LSPR signals. Furthermore, controlling the thickness of the PANI layer shares an identical underlying mechanism and effectively shifts the color dynamic range, providing striking dynamic color palettes in scattering, transmission, and reflection. However, increasing the PANI thickness strengthens its imaginary refractive index, extinction ($k$), leading to visibly darker images in scattering and transmission (e.g., Fig. 4a, c)^45^. Hence, we identify a threshold for PANI thickness (21 nm) within this composite structure, allowing for optimal multicolor filtering and providing the broadest modulation wavelength range with superior optical contrast.

**Fig. 5:**
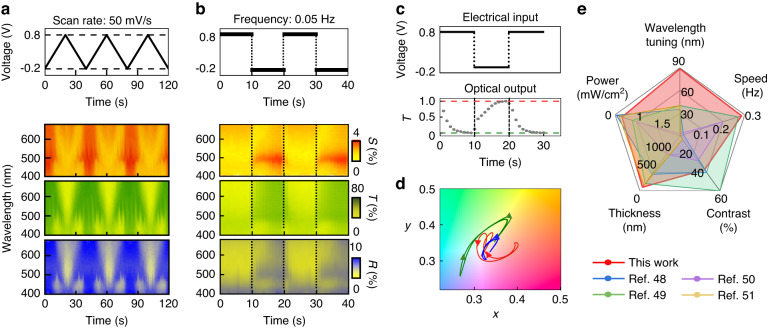
Electrical and optical switching dynamics of the dichroic multicolors. **a** CV of the applied potential (top) and corresponding optical switching in scattering, transmission, and reflection (bottom). **b** Step modulation of the applied potential at 0.05 Hz (top), and corresponding reversible optical switching (bottom), and **c** single cycle operation for the transmission switching at the peak wavelength of 480 nm with 3.5 s of transient time. **d** Associated color gamut plots of the spectra in (**a**, **b**). **e** Performance comparison with devices in the literature^48–51^

We then evaluate the device’s switching stability and speed through two modulation approaches (Fig. 5 and Video S3). First, we operate the device with cyclic voltammetry spanning a potential range of −0.2 V to 0.8 V, performed over 3 cycles (Fig. 5a). Throughout this cycling, scattering, transmission, and reflection modes consistently exhibit remarkable color dynamics, each presenting degradation-free transitions over the course of 30 cycles (Fig. S8). Second, we introduce step-function voltage transitions oscillating between ${V}_{R}$ and ${V}_{O}$ at frequencies of 0.05 Hz, 0.5 Hz, and 2.5 Hz (Fig. 5b for 0.05 Hz, Fig. S9 for 0.5 Hz & 2.5 Hz). This controlled modulation clearly shows swift color switching across all modes of scattering, transmission, and reflection with no frequency delay. Furthermore, the analysis of the rising and falling optical transitions in transmission mode provides the rising and falling switching times, quantified at 3.4 s and 1.7 s, respectively (Fig. 5c). These prolonged and differential transition times stem from the proton doping and dedoping process, essentially involved in the redox reaction of PANI^46^; this can be further reduced by engineering the physical dimension and ion conductivity of the electrolyte^47^. We also confirm a robust correlation in color switching efficiency across all three modes: reflection, scattering, and transmittance through the optical images and CIE 1931 color space (Fig. 5d). Hence, this active dichroic multicolor switching demonstrated here appears highly competitive with current adaptive filter technologies (Fig. 5e)^48–51^. In particular, the key features include visible wavelength tuning (reaching a 100 nm shift), ultrasmall thin architecture (55 nm), and fast switching (ca. 0.3 Hz): these feature have been previously difficult to achieve. Notably, this device shows a maximum of 40% optical contrast at resonance with power densities below 200 μW/cm^2^; these are sufficient for device application, but these performances can be further improved by optimizing the structural geometry of the plasmonic nanocomposites, e.g., creating a multilayer of the Au NPs with reduced PANI thickness.

### Warm-to-cool color temperature switching

**Fig. 6:**
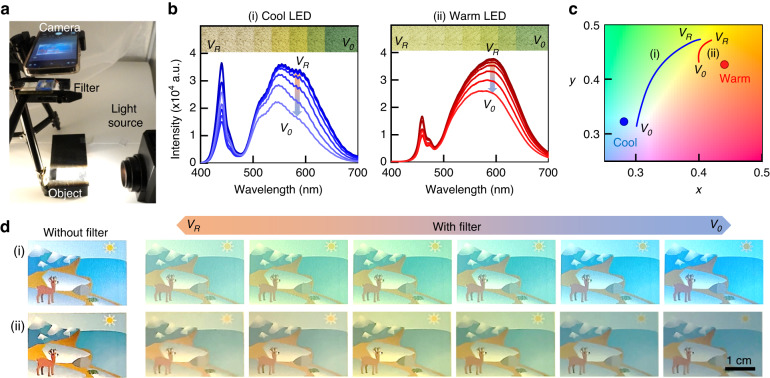
Warm-to-cool color temperature switching. **a** Experimental setup. **b** Color changes in the transmission through the plasmonic nanofilter when illuminated by (i) cool (left) and (ii) warm white LEDs (right) and **c** color gamut (CIE 1931). **d** Images of the tangible objects with various color temperatures modulated by the nanofilter under (i) cool (top) and (ii) warm white LEDs (bottom)

To enable our plasmonic nanocomposite as a prospective tool for adaptive multicolor filtering, we systematically investigated its transmission characteristics under different lighting conditions, including cool and warm white LED sources (Fig. 6 and Video S4). The cool white LED emits a pristine white hue with a color temperature of 6500 K (coordinates of 0.28, 0.32 in the CIE 1931 plot), while the warm white LED produces warmer yellow tones at a color temperature of 3000 K (coordinates of 0.44, 0.43 in the CIE 1931 plot). For a comprehensive evaluation, we perform measurements on the light spectrum through our filter without reference processing (Fig. 6a). When subjected to illumination by the cool white LED, the transmitted light exhibits a noticeable color shift, transitioning from white to yellow and blue (left panel of Fig. 6b). This shift is attributed to 75% suppression at the 555 nm wavelength based on the switching fidelity of the plasmonic nanofilter. Upon implementing the warm white LED light source, the transmitted light changes from yellow to white, reflecting associated color temperature changes from 6250 K to 4000 K (right panel of Fig. 6b). As shown in Fig. 6c, a noticeable convergence occurs between the color coordinates of the cool and warm white LEDs, indicating the filter’s capacity to switch color temperature effectively from 3250 K to 6250 K with a 1 V operating voltage sweep. Furthermore, to validate the consistent color perception of tangible objects under each light source, the objects are observed through the nanofilter, with close examination of the performance of the adaptive dichroic multicolor filter (Fig. 6d). Remarkably, the experimental results show object images with various color temperatures under both cool and warm white LED lighting conditions. Thus, the plasmonic nanocomposite filter strongly demonstrates its filtering capability across diverse light temperature conditions; this can be extended to various light-shifting scenarios, spanning from day to night, different geographical locations, and varying weather conditions.

## Discussion

In summary, we demonstrate an electrically tunable dichroic multicolor filter exhibiting bidirectional full-color dynamics. The wafer-scale thin film (below 100 nm) of the plasmonic nanocomposite filled with an electrochemically active medium of polyaniline enables impressive multicolor dynamics across the entire visible spectrum. This exceptional modulation shows approximately 66 nm (green to red) in scattering, 89 nm (blue to yellow) in transmission, and 42 nm (blue to green) in reflection. Remarkably, this dynamic modulation needs minimal voltage input (below 1 V) while exhibiting rapid but degradation-free switching capabilities, as evidenced by the transition times of 3.4 s. This switching fidelity enables the mimicking of the adaptive color control in the eyes of an arctic reindeer as a form of dichroic multicolor filter. As a proof of concept, we successfully manipulate the color temperature, shifting from warm to cool tones and effectively spanning from 3250 K to 6250 K; this is accomplished through the application of a single plasmonic nanofilter. This dynamic color calibration at the device level not only aligns with and complements the emergent domain of the machine learning-driven object recognition systems^2^ but also has potential for enhancing a myriad of electronic devices, encompassing both personal items and public management systems^52,53^.

## Materials and methods

### PANI electrodeposition

PANI is synthesized through an electrodeposition process onto the surface of an ITO substrate^54^. A total of 1.5 mL of 100 mM aniline dispersed in 2 M HNO_3_ is injected into the electrochemical cell where the 3 electrodes are all connected. The ITO substrate serves as the working electrode for electrochemical-assisted PANI polymerization. Pt mesh is used as a counter electrode with an Ag/AgCl reference electrode. The potentiostat runs cyclic voltammetry with a potential range of 0 V to 1.05 V at a scan rate of 20 mV/s, leading to the growth of the PANI layer at a rate of 0.8 nm per cycle.

### Au NP growth

Au NPs are grown on the PANI-coated ITO substrate using an electron evaporation method with the substrate cooling at 237 K and a base pressure of 5 × 10^–6 ^Torr at a rate of 0.5 nm/s. The deposition thickness is controlled from 2 nm to 5 nm with a step size of 1 nm.

### SEM analysis

SEM images of the samples were obtained using a Hitachi S-4700 scanning electron microscope (Hitachi) at an accelerating voltage of 10 kV. To analyze the NP size, we measured the area of each Au NP using ImageJ software and calculated the fill fraction and effective diameter ($2{r}_{e}$) of the Au NPs by assuming that the Au NPs were spherical and calculating their diameter with the following formula: ${A}_{{measured}}=\pi {r}_{e}^{2}$.

### Optical microscopic imaging and spectroscopy with electrochemistry

Optical images and spectra of the samples were obtained using an Omron Sentech STC-MCS500U3V camera and spectrometer (Ocean Optics QE-PRO) with a 5$\times$ objective (Olympus MPLFLN5xDB; numerical aperture: 0.15) in a microscope (Olympus GX53). The light sources are cool and warm white LEDs (Philips 409850, Philips Fortimo SLMC830). To measure optical signals while operating electrochemistry, the ITO substrate of the sample serves as the working electrode, a Pt mesh acts as the counter electrode, and all three electrodes are inserted into a fluid chamber; this chamber is created by adhering two clean glasses with a stack of double-sided tape and filling with the electrolyte solution (0.5 M NaCl in 10 mM HCl). The refractive index of PANI is adjusted using cyclic voltammetry within a potential range of −0.2 V to 0.8 V (vs. Ag/AgCl reference electrode) at a scan rate of 50 mV/s.

### Numerical simulation

Optical responses of the plasmonic nanocomposite are simulated using commercial software (Ansys Lumerical solution). Au NPs with a size of 11 nm are embedded within a 30 nm thick PANI layer placed on a 120 nm ITO thin layer. Au NPs are randomly distributed with a fill fraction of 25% by using the component embedded in the software, called a ‘uniformly random distribution.’ The incident light is illuminated as a plane wave polarized perpendicular to and propagating toward the substrate for the calculations of reflection and transmission. Their values are calculated by integrating the total power flow, $\vec{P}$, across the observing surface, $S$, with the following equation: ${Power}\left(\omega \right)=\frac{1}{2}\int {real}\{\vec{P}(\omega )\}\cdot d\vec{S}$. For scattering, a total-field scattered-field source is used with the same polarization and propagation vector. The optical properties of Au, PANI, ITO, and glass were obtained from the literature^16,55^. The surrounding refractive index was set to *n* = 1.33 to match the experimental environment.

### Supplementary information

Video S1

Video S2

Video S3

Video S4

Supporting information
