# Supplementary material for: Programmable directional color dynamics using plasmonics
Source: Microsyst Nanoeng. 2024 Feb 1;10:22. doi: 10.1038/s41378-023-00635-8 (PMC10831043; doi:10.1038/s41378-023-00635-8)
Supplement: Supplementary file 5 — Supporting information [file 41378_2023_635_MOESM5_ESM.docx]

Supporting Information

**Programmable directional color dynamics using plasmonics**

Gyurin Kim,^1^ Doeun Kim,^1^ Soeun Ko,^1^ Jang-Hwan Han,^1^ Juhwan Kim,^1^ Joo Hwan Ko,^1^

Young Min Song,^1,2,3,*^ Hyeon-Ho Jeong^1,2,*^

^1^ School of Electrical Engineering and Computer Science, Gwangju Institute of Science and Technology, Gwangju 61005, Republic of Korea

^2^ Department of Semiconductor Engineering, Gwangju Institute of Science and Technology, Gwangju 61005, Republic of Korea

^3^ Artificial Intelligence (AI) Graduate School, Gwangju Institute of Science and Technology, Gwangju 61005, Republic of Korea

^*^Corresponding author: ymsong@gist.ac.kr, jeong323@gist.ac.kr

**Keywords**: active plasmonics, metasurfaces, multicolor filters, programmable dichroism

**Video S1.** Electrically tunable scattering, transmission, and reflections of the plasmonic nanocomposites.

**Video S2.** Programmable multicolors of the plasmonic nanocomposites with PANI thickness engineering.

**Video S3.** Optical switching dynamics of the plasmonic nanocomposites at 0.1 Hz frequency.

**Video S4.** Warm-to-cool color temperature switching using the plasmonic nanocomposites.


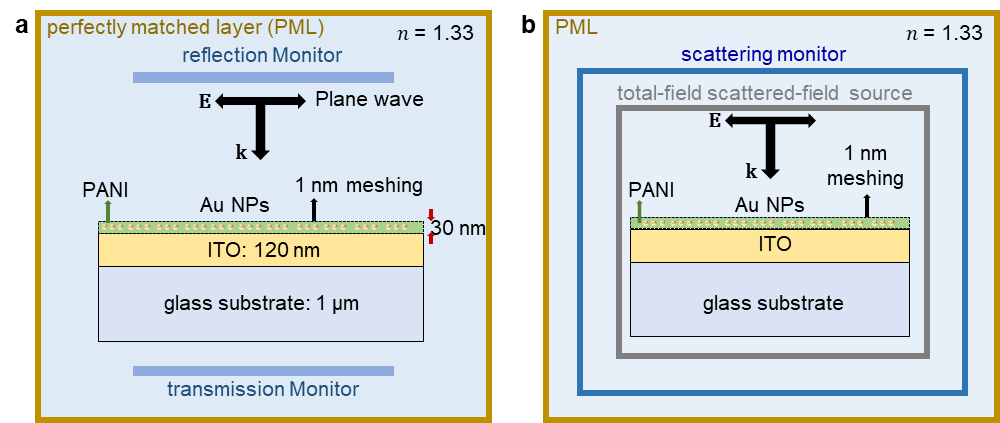


**Figure S1** (a) Modellings of plasmonic nanocomposite to numerically simulate (a) reflection and transmission and (b) scattering.


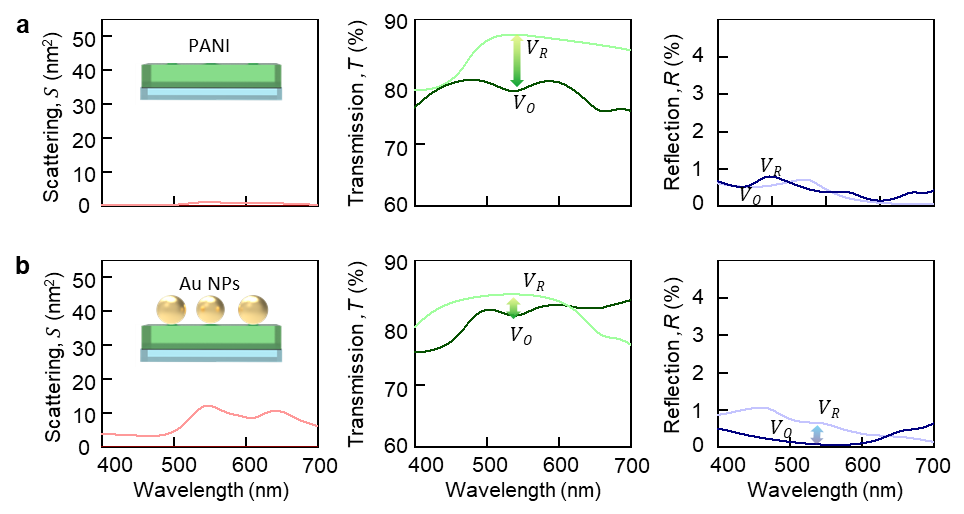


**Figure S2** (a) Far-field scattering (left panel), transmission (middle panel), and reflection (right panel) of the PANI layer and (b) those of Au NPs on the PANI layer when the applied voltage changes between $V_{R}$ (voltage for fully reduced PANI) and $V_{O}$ (voltage for fully oxidized PANI) from numerical simulations.


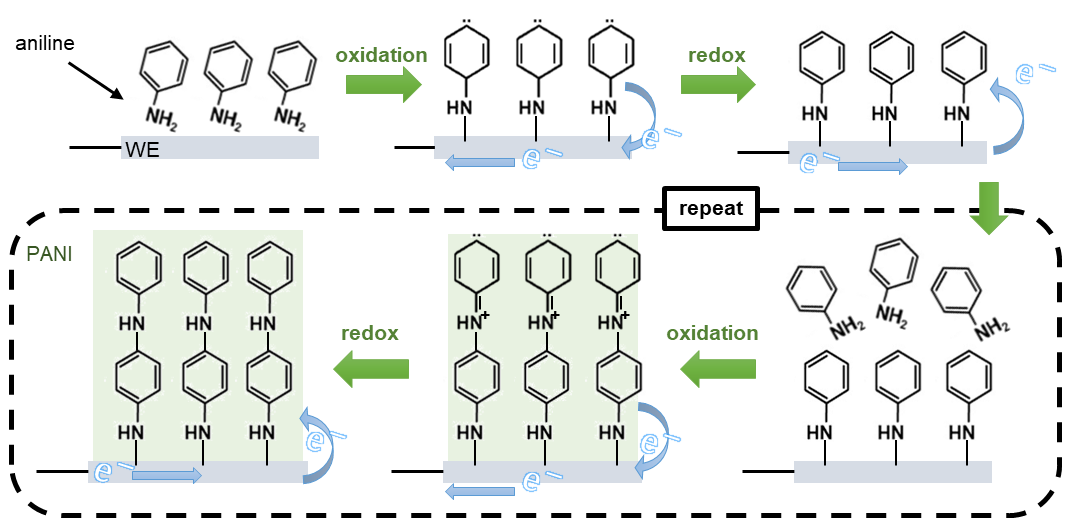


**Figure S3**. Electrodeposition process of the PANI layer from aniline monomer.


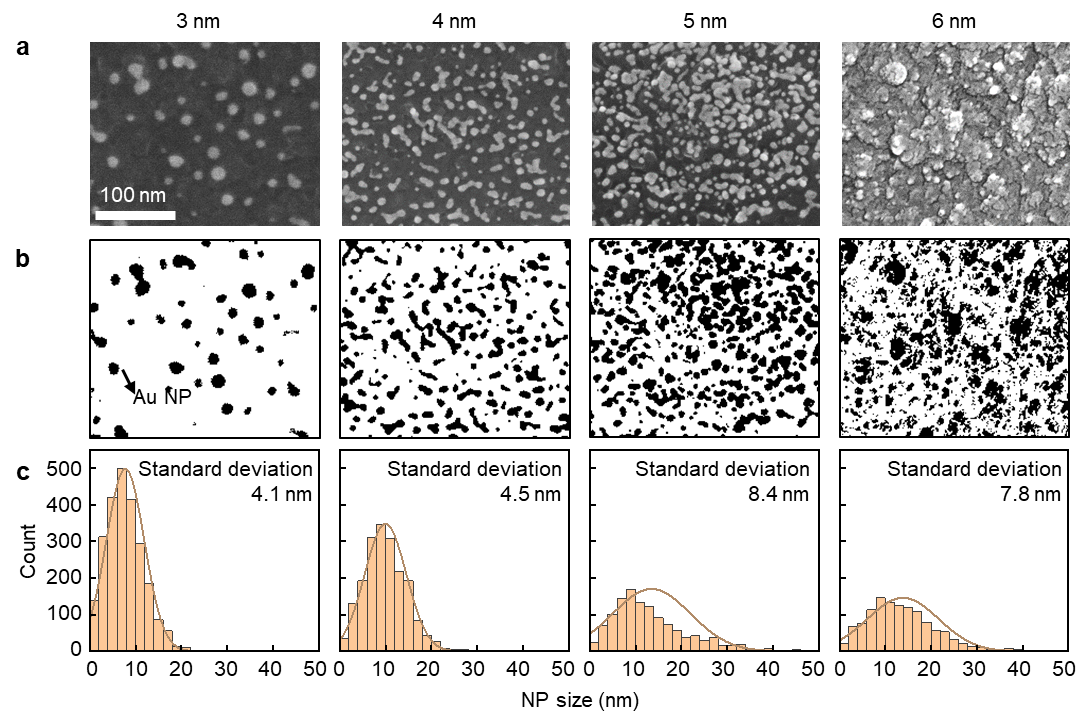


**Figure S4**. (a) SEM images of the Au NPs on the PANI layer, achieved from different growth thicknesses, ranging from 3 nm to 6 nm with 1 nm intervals (from left to right). (b) Their color-saturated images and (c) measured size distributions.


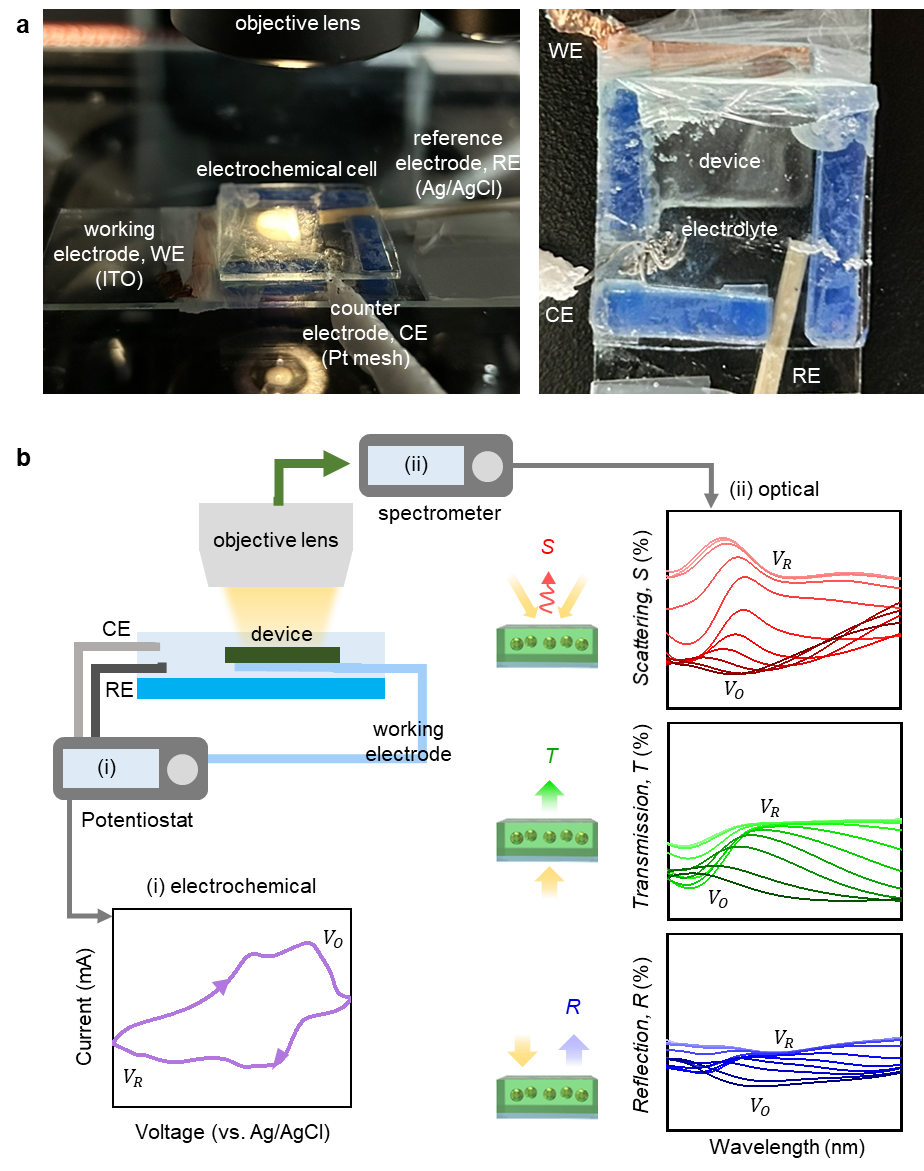


**Figure S5**. (a) The electrochemical cell assembled with optical and electrochemical measurement systems. (b) Schematic of the experimental setup with different optical measurement modes.


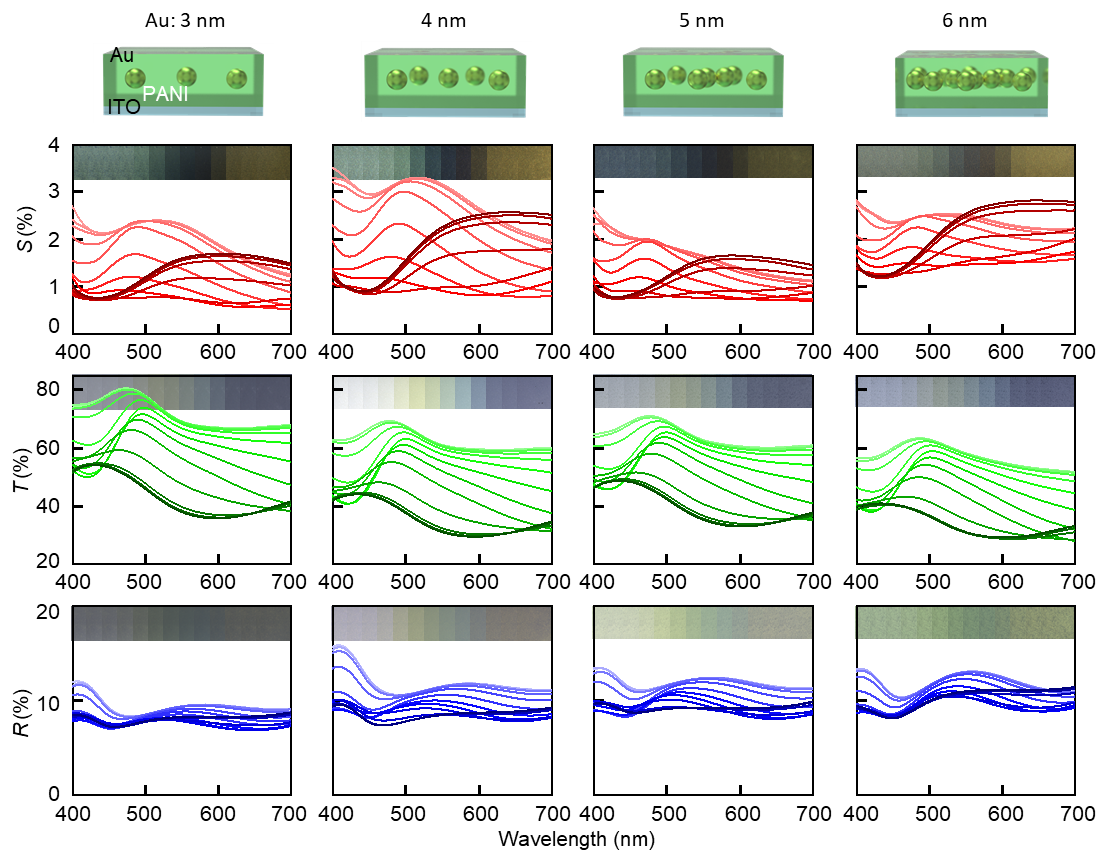


**Figure S6**. Experimentally measured color dynamics of scattering (top), transmission (middle), and reflection (bottom) with different Au growth thickness from 3 nm to 6 nm (from left to right) when the voltage is applied from -0.2 V ($V_{R}$) to 0.8 V ($V_{O}$). Each inset is corresponding optical images.

**
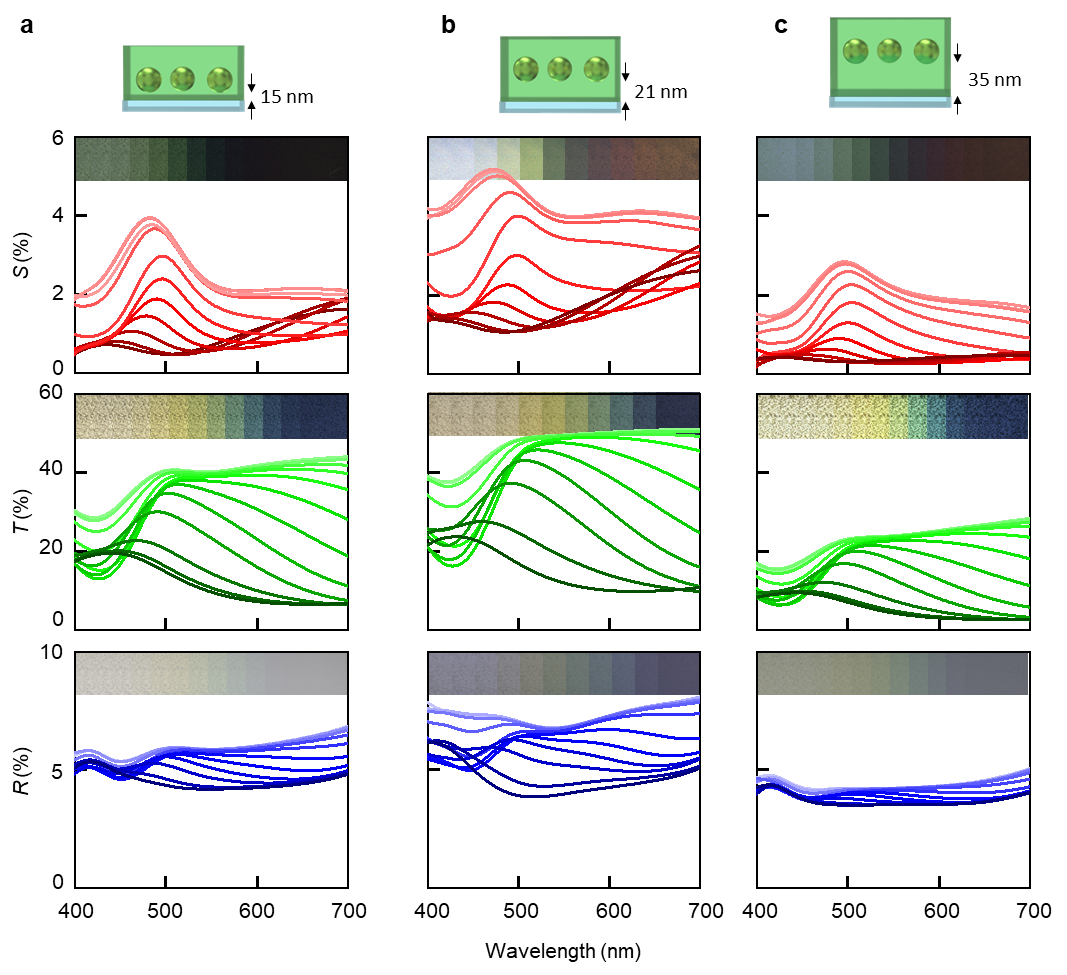
**

**Figure S7**. Experimentally measured color dynamics of scattering (top), transmission (middle), and reflection (bottom) of the 11 nm Au NPs embedded within the PANI layer where the bottom PANI layer thickness changes from (a) 15 nm (b) 21 nm, to (c) 35 nm. The voltage is applied from -0.2 V ($V_{R}$) to 0.8 V ($V_{O}$). Each inset is corresponding optical images.


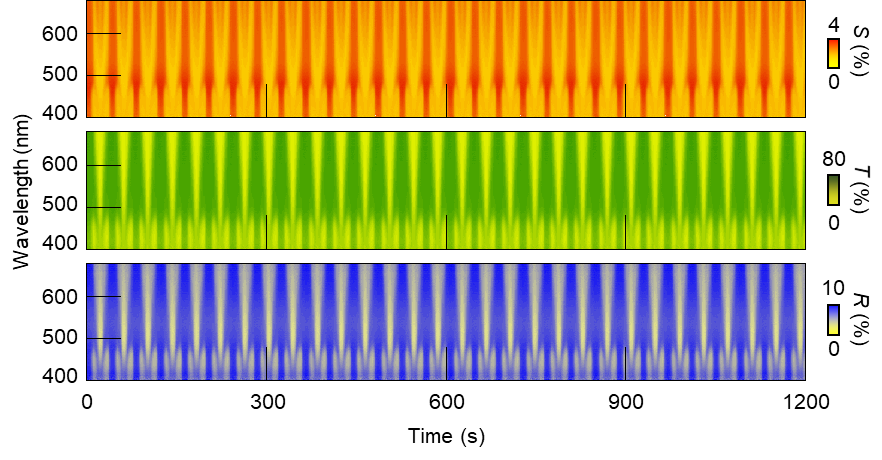


**Figure S8**. Experimentally measured color dynamics of scattering (top), transmission (middle), and reflection (bottom) of 11 nm Au NPs embedded within the PANI layer when the voltage is applied from -0.2 V ($V_{R}$) to 0.8 V ($V_{O}$) over 30 cycles.


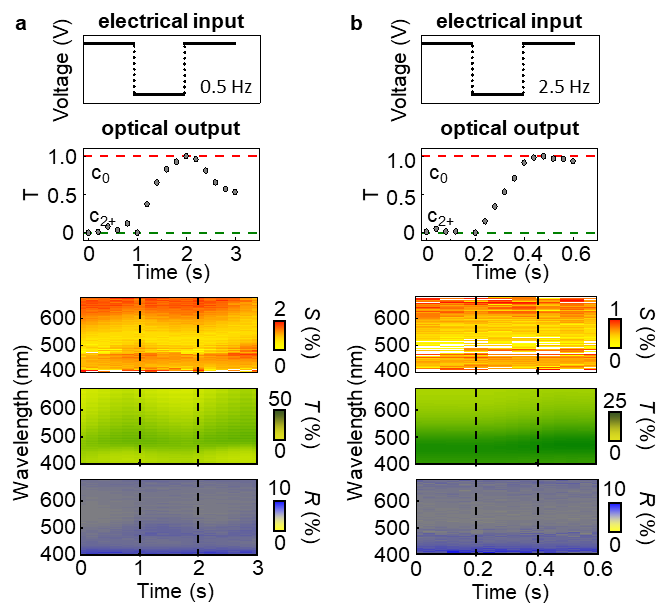


**Figure S9**. Step modulation of the optical signals of the plasmonic nanofilter with (a) 0.5 Hz and (b) 2.5 Hz.
